# Mental toughness and choking susceptibility in athletes

**DOI:** 10.3389/fpsyg.2024.1414499

**Published:** 2024-07-22

**Authors:** Burgandy Thiessen, Mishka Blacker, Philip Sullivan

**Affiliations:** Faculty of Applied Health Sciences, Brock University, St. Catharines, ON, Canada

**Keywords:** choking susceptibility, mental toughness, athletes, self-consciousness, anxiety, coping styles

## Abstract

Choking susceptibility refers to the propensity of an athlete to choke under pressure. Mesagno has operationalized choking susceptibility as a combination of scores on self-consciousness, anxiety and coping. Despite the potential of Mesagno’s protocol, there is currently limited support for its validity. Secondly, although mental toughness (MT) has a relationship with sport performance, there is limited research on its relationship to choking under pressure, specifically. The current study investigated the relationship between choking susceptibility and mental toughness. It was hypothesized that choking susceptible athletes will have significantly lower levels of mental toughness than those who are not choking susceptible. Data from a heterogeneous sample of athletes (*N* = 415) was obtained through a Qualtrics research panel. Results of a Mann–Whitney U showed that self-reported mental toughness was not significantly different in athletes categorized as choking susceptible and non-choking susceptible. Correlational analyses also highlighted differences between mental toughness and the composite scores of choking susceptibility, which provide researchers with avenues for future research in this area alongside a need for each construct to be examined in relation to choking behavior in sport.

## Introduction

Success and failure are often dependent on an individual’s ability to effectively perform under heightened levels of pressure. Experiencing pressure can affect how an individual performs an otherwise automatic motor task (Geukes et al., 2012; Mesagno et al., 2019; Roberts et al., 2019). For some, the pressure can often become overwhelming and result in what is known as choking under pressure (referred to as choking hereafter). Historically, there have been issues with the definition of choking (Hill et al., 2010). The initial definition of choking by Baumeister (1984, p. 610) was ‘performance decrements under pressure situations’ and more specifically, ‘the occurrence of inferior performance despite striving and incentives for superior performance’ (Baumeister and Showers, 1986, p. 361). Some researchers have argued that such definitions may fail to reflect the entire choking experience (e.g., Gucciardi and Dimmock, 2008). For example, Beilock and Gray (2007) suggest that in order for a sub-optimal performance to be considered a choke, it must be certain that the athlete was motivated to achieve their goal, was capable of performing better, and regarded the situation as important. A choke is not a random fluctuation in skill level, but rather a specific negative response to perceived pressure (Hill et al., 2010). The sources of ‘perceived pressure’ typically include spectators, evaluation, rewards, skill level, perceived importance, and time constraints (Cao et al., 2011; Murayama and Sekiya, 2015). These sources evoke cognitive and behavioral reactions such as distraction, self-consciousness, and anxiety, which can induce the phenomenon of choking. The existence of choking highlights the fragility of expert performance within an individual, demonstrating that constant and consistent execution in training does not guarantee skilled performance in crucial moments. Many factors have been found to influence an individual’s likelihood of choking. For example, anxiety (Wilson, 2008; Clarke et al., 2020), perfectionism (Frost and Henderson, 1991; Yoon et al., 2021), and fear of negative evaluation (Mesagno et al., 2012), can interfere with one’s ability to perform under pressure. Additionally, feelings of physical fatigue and heaviness, abnormal physical sensations, ego relevance, and changes in motor control have been related to an increased probability of choking (Wang, 2002; Murayama and Sekiya, 2015).

Choking susceptibility is the likelihood that an individual will experience choking (Mesagno et al., 2008). Mesagno et al. (2008, 2009) developed a protocol for identifying choking susceptibility in athletes, which is rooted in specific levels of self-consciousness (Self-Consciousness Scale; SCS; Fenigstein et al., 1975), anxiety (Sport Anxiety Scale; SAS; Smith et al., 1990), and coping styles (Coping Style Inventory for Athletes; CSIA; Anshel and Kaissidis, 1997). This protocol is currently the only method available to assess choking susceptibility, which classifies athletes as choking susceptible or choking non-susceptible based on their relative scores on these three attributes. To determine if one is choking susceptible, participants must score within the 75th-100th percentile range on at least two out of three choking susceptible inventories (based on the sample of individuals tested), and their remaining score must fall within the 50th-100th percentile range of scores surveyed. For example, to be considered choking susceptible, the participant could be relatively high (i.e., in the 75th-100th percentile) in self-consciousness (SCS) and trait anxiety (SAS), and have a positive differential CSIA score (i.e., approach coping – avoidance coping = differential score). Combining these scales to inform athletes’ level of choking susceptibility has been used in previous research by Mesagno et al. (2008, 2009), Wang et al. (2004a,b), and with non-athletes (Thiessen et al., 2023). Importantly, being classified as choking susceptible does not guarantee that an athlete will choke (or that an athlete classified as non-susceptible will never choke), but rather, suggest a higher likelihood of that event occurring.

Trait anxiety may be the most obvious predictor that Mesagno et al. (2008) included in their protocol, since anxiety has consistently been linked to poor attention selection and performance (Woodman and Hardy, 2003). Anxiety research has confirmed that individuals high in trait anxiety react to pressure situations with greater levels of state anxiety than individuals low in trait anxiety (e.g., Spielberger et al., 1976; Horikawa and Yagi, 2012). It has been reported that this effect happens because high trait anxious individuals respond to pressure with elevated state anxiety more frequently or intensely, which ultimately affects their performance under pressure (Byrne and Eysenck, 1995). Furthermore, high trait anxiety overwhelms working memory which causes inefficient processing and promotes choking (Wilson et al., 2007). Likewise, high trait anxious athletes are susceptible to choking via self-focus mechanisms as they also tend to have high dispositional reinvestment (Masters et al., 1993). Similarly, coping ability is consistently found to influence performance under pressure in sport (Anshel, 1996; Wang et al., 2004a; Nicholls and Polman, 2007). Avoidance-based coping (i.e., directing activity away from threat) has been found to be a beneficial coping style under stress (Madden et al., 1990; Anshel, 1997). Alternatively, approach-based coping (i.e., directing activity toward threat) can result in the performer consciously controlling behavior during stress, which is known to decrease performance and result in a choke (Baumeister, 1984; Wang et al., 2004a). Consistent with this conceptualization, Diotaiuti et al. (2021) found that an approach coping style appeared to have the greatest negative effect on archer athletes brooding activity. Lastly, self-consciousness is known to be negatively related to performance. In a series of studies, Geukes et al. (2013a,b) found that self-consciousness negatively affected performance in high pressure conditions, and that lower self-consciousness was related to better performance in high pressure conditions, but not low-pressure conditions. Wang et al. (2004b) found that self-conscious athletes were more susceptible to choking.

Since Mesagno et al. (2008) defined choking susceptibility, researchers have examined linkages between this conceptualization and other constructs. For example, Mesagno and Marchant (2013) investigated the cognitive processes associated with choking susceptible and choking resistant athletes in a mixed-method approach. The authors designed a pressured task (i.e., netball shots with audience presence, performance-contingent financial incentives, and video recorded shot attempts), and reported that the cognitions associated with choking susceptibility included emotion-focused attention (e.g., fear, embarrassment, worry) and approach-cognitive coping strategies (e.g., public self-awareness), psychodynamic defense mechanisms (i.e., projection), whereas choking resistance was associated with task-focused attention (e.g., self-talk) and avoidance-cognitive coping strategies (e.g., blocking out distractions, imagining team support). Additionally, those considered to be choking susceptible experienced a decrease in performance (i.e., less accurate and more inconsistent) during the pressure manipulation compared to those who were considered choking resistant. Choking susceptibility has also been found to be related to handedness (Mesagno et al., 2019), dominant left-hemispheric activation (Hatfield et al., 2013), and personality traits (e.g., neuroticism, perfectionism, etc.; Frost and Henderson, 1991; Thiessen et al., 2023). Mesagno et al.’s choking susceptibility protocol is the only procedure available to measure choking susceptibility to date.

Empirical evidence has linked choking susceptibility with various outcomes that are both related to performance under pressure and consistent with the conceptual foundations for Mesagno et al.’s (2008) operational definition of the construct. However, more evidence is needed to support choking susceptibility’s concurrent and predictive validity since surprisingly, there is little evidence available that it is related to choking. The current study aims to further support the concurrent validity of choking susceptibility by examining how it is related to mental toughness.

Mental toughness is a term typically applied to athletes who perform well in pressurized circumstances (Liew et al., 2019; Bédard Thom et al., 2021). It has been called a “critical success factor” (Cowden, 2017, p. 1) because it is believed to facilitate adaptive responses to positive (e.g., success) and negative (e.g., failure) forms of stress (e.g., competition pressure). According to Gucciardi et al. (2017), mental toughness is “a state-like psychological resource that is purposeful, flexible and efficient in nature for the enactment and maintenance of goal directed pursuits” (p. 18). Although debate surrounding the conceptualization of mental toughness continues (i.e., stability, different types) much of the research to date supports that it is a malleable, psychological quality that is greatly dependent on self-belief/confidence and perceived self-control in the face of challenges and stressors (Bédard Thom et al., 2021). Excellent coping strategies and perseverance would appear to link mental toughness with successful performance (Bell et al., 2013; Gucciardi et al., 2016; Cowden, 2017; Lin et al., 2017; Giles et al., 2018; Gucciardi et al., 2021). For example, Bell et al. (2013) developed and delivered a mental toughness intervention for elite cricket players that enhanced their ability to perform under pressure, specifically in their batting and fitness performance. There is also evidence suggesting that athletes with higher mental toughness can more quickly initiate performance enhancing states (i.e., flow and clutch states) during sport competition (Jackman et al., 2020).

Given that mental toughness is associated with excelling under pressure, theoretically, mental toughness could be related to choking susceptibility. Hill and colleagues published a series of studies involving the nature of mental toughness relative to choking. A focus group of sport psychologists concluded that lower mental toughness is linked to the probability of choking, and that sport psychologists should prioritize its development to reduce the probability and impact of choking (Hill et al., 2009). Another study by Hill et al. (2010) purposefully sampled elite golfers (*N* = 6) who believed they “often choked under pressure,” compared to golfers who appeared to “excel under pressure” (*N* = 5), as well as elite coaches who had worked with both groups (*N* = 4). Following semi-structured interviews, the authors found that that those who were more prone to choking appeared to have lower mental toughness than those who were categorized as golfers who excel under pressure (Hill et al., 2010). However, mental toughness was not directly assessed in these studies, and prevalence of choking was based on participants’ self-perceptions, not Mesagno et al.’s (2008) operational definition or measured choking behavior.

According to Mesagno’s protocol, using more avoidance than approach strategies will work to prevent choking under pressure (Wang et al., 2004a). However, research has shown that, for the most part, avoidance coping strategies are associated with choking under pressure and approach strategies may encourage clutch performance (Hill and Hemmings, 2015). For example, Hill et al. (2010) explains that chokers predominantly use avoidance strategies (e.g., rushing through shots) to cope, however, this is not the case for those who excelled; they tended to reduce or manage the stressors through problem-focused or approach coping (e.g., process goals and preparation). This discrepancy could in part be due to the different instruments used in the coping literature but may also be the result of avoidance strategies offering individuals immediate emotion regulation in the short term, which can encourage positive behavioral outcomes (e.g., Hayes et al., 1996) but are less effective in the long term (Hill and Hemmings, 2015). In terms of the relationship between coping and mental toughness, Nicholls et al. (2008) found that mental toughness was more strongly associated with the use of approach coping strategies (i.e., task-oriented coping strategies such as thought control, mental imagery, relaxation, effort expenditure, logical analysis, and seeking support) compared to avoidant strategies (i.e., distraction-orientated coping such as distancing and mental distraction, or disengagement-orientated coping such as disengagement/resignation and venting of unpleasant emotions) in athletes. Madrigal et al. (2017) found that mentally tougher collegiate athletes reported the use of more problem and emotion focused coping strategies rather than avoidant coping strategies. To our knowledge, no research has assessed the relationship between mental toughness and scores on the CSIA used in Mesagno’s protocol. Self-consciousness (and the related concept of self-awareness) have also been linked to mental toughness. Mentally tougher competitive tennis players have been found to have greater levels of self-awareness (Cowden, 2017), and researchers have found that mental toughness is related to dispositional flow which includes an ability to lose consciousness aware (i.e., concern for the opinion of others disappears; Crust and Swann, 2013; Jackman et al., 2017). Consistent with these findings, the pressure of being watched by others (a common manipulation to increase performance pressure) increases self-consciousness and self awareness (DeCaro et al., 2011).

The relationship between mental toughness and anxiety is somewhat inconsistent, yet self-belief and self-confidence is the most commonly reported psychological attribute associated with athlete mental toughness (e.g., Gucciardi et al., 2015; Bédard Thom et al., 2021). Athletes can experience anxiety when they lack confidence or self-efficacy in their ability to perform successfully in a threatening or taxing situation; they believe that they are incapable of managing potentially detrimental events (Chase et al., 2005). Thus, mentally tougher athletes are expected to report somewhat lower levels of sport trait anxiety (Mojtahedi et al., 2023), which will expectedly contribute to lower choking susceptibility.

The purpose of this study was to determine if there will be a difference between choking susceptible and choking non-susceptible athletes’ level of mental toughness. Based on conceptualizations of mental toughness and choking susceptibility, and research linking them to performance under pressure, we hypothesized that choking susceptible athletes will report lower levels of mental toughness than choking non-susceptible athletes.

## Method

### Participants

Using Qualtrics, athletes across Canada and the United States of America were recruited to participate in the online study. We requested our target audience (i.e., athletes in North America above the age of 18 years old) from Qualtrics who found a representative sample from their proprietary online sample. Qualtrics completed all recruitment and distribution of the survey to participants. All data was stored on Qualtrics’ secure platform during collection. Once data was collected, Qualtrics sent us a data scrub report where they outlined what participants could be removed due to lack of data, straightening, etc. Final removal of participants was ultimately made by the researchers. The questionnaires were blocked into separate pages throughout the survey for participant ease of use and items of each questionnaire were presented in a matrix. For inclusion to participate, individuals must have been 18 years or older and participate in a sport. No other inclusion or exclusion criteria were applied. Participants were compensated through the vendors who partner with Qualtrics. Participants agree upon a set compensation before taking part in the survey which could be in the form of points, airline miles, etc.

We obtained a total of 425 responses and of that total, 10 were removed due to straightlining and lack of sport clarification. Therefore, the final sample size was 415 participants, with a total of 187 females, 224 males, 3 non-binary/third gender, and 1 participant that preferred not to indicate gender. Participants’ age ranged from 18–80, with an average age of 40.56. Only 316 valid responses for age were given. Competitive athletes were defined by those who indicated they participated in international (*n* = 9), national (*n* = 28), provincial/state (*n* = 53), university/college (*n* = 45), and intermediate (*n* = 72) levels of sport. Participants were from 39 different sports. The most common sports included basketball (*n* = 91), soccer (*n* = 40), football (*n* = 34), tennis (*n* = 32), golf (*n* = 30), softball (*n* = 24), baseball (*n* = 23), and volleyball (*n* = 18). Additionally, participants were asked to indicate whether or not they were starters in their respective sport; 79.4% reported being a regular or occasional starter.

### Procedure

Prior to recruitment and data collection, ethical clearance was granted by Brock University’s Research Ethics Board 21–274. Data was collected between July–August 2022. The choking susceptibility protocol comprises the SCS (Fenigstein et al., 1975), the SAS (Smith et al., 1990), and the CSIA (Anshel and Kaissidis, 1997).

### Measures

Questionnaires measured participant demographic information, mental toughness, and choking susceptibility. Demographics included questions regarding gender, age, ethnicity, and athletic status. Choking susceptibility was determined using a combination of measures examining self-consciousness, trait anxiety, and coping styles. A unidimensional measure was used to assess sport mental toughness.

#### Self-consciousness scale

The 23-item Self-Consciousness Scale (Fenigstein et al., 1975) measures three distinct subscales of self-consciousness (i.e., private self-consciousness, public self-consciousness, and social anxiety). Items are rated on a scale of 0 (*extremely uncharacteristic*) to 4 (*extremely characteristic*) where those with higher scores report higher levels of public self-consciousness, private self-consciousness, and social anxiety. Acceptable internal consistency (*α* > 0.73) has been reported for all subscales (Fenigstein et al., 1975). In a sample of athletes, the public self-consciousness subscale had a Cronbach’s alpha of 0.75, private self-consciousness had a Cronbach’s alpha of 0.70, and social anxiety had 0.80 (Hatzigeorgiadis, 2002); Cronbach’s alpha for the scales global factor, which was used for this protocol, was 0.84 with the current data. There is also considerable evidence for both the construct and discriminant validity of the distinct subscales of self-consciousness (Fenigstein, 1987).

#### Sport anxiety scale

To assess trait anxiety, the 21-item Sport Anxiety Scale (Smith et al., 1990) was used. The SAS is made up of three subscales that specifically measure somatic anxiety, worry, and concentration disruption. Statements and responses are based on a 4-point Likert scale, ranging from 1 (*not at all*) to 4 (*very much so*). Total scores range from 21 to 84, with higher scores indicating high trait anxiety. The SAS has shown good internal consistency results and adequate validity in athletes (Smith et al., 1990; Dunn et al., 2000). The Cronbach’s alpha for the total scale was 0.96 with the present data.

#### Coping style inventory for athletes

The Coping Style Inventory for Athletes (Anshel and Kaissidis, 1997) is a 16-item questionnaire used to measure participants’ approach and avoidance coping strategies on a 5-point Likert scale. Responses range from 1 (*very untrue*) to 5 (*very true*). Total scores range from 8 to 40 on each of the two subscales, and higher scores indicate a greater propensity to use that particular coping style. High construct and predictive validity have been reported for the scale, as well as acceptable internal consistency in a sample of athletes (Kaissidis-Rodafinos et al., 1997); the current data showed Cronbach’s alphas ranging from 0.67–0.76. For the choking susceptibility protocol, the differential score is calculated by taking the total avoidance coping score and subtracting it from the total approach coping score (e.g., Mesagno et al., 2008).

#### Mental toughness index

The Mental Toughness Index (MTI; Gucciardi et al., 2015) is an 8-item unidimensional measure of mental toughness.^1^ The MTI instructs participants to indicate how they typically think, feel, and behave as an athlete. The MTI is rated on a 7-point Likert scale (*false, 100% of the time; true, 100% of the time*). The MTI was intentionally developed to bring together the most common attributes of mental toughness across the field, and to be conceptually distinct from other similar constructs that are also known to be influential on sport performance such as Grit, Resilience and Hardiness (Gucciardi et al., 2015). The MTI has also demonstrated cross-cultural invariance in athlete samples (Stamatis et al., 2021). In support of the MTI’s construct validity, Gucciardi et al. (2015) demonstrated excellent fit using CFA across three independent samples that were purposefully selected to represent different achievement contexts (i.e., athletes, post-secondary students, and “white collar” workers). They also reported excellent composite reliabilities for the scale in these samples (*ρ* = 0.86 to 0.89). Cronbach’s alpha with the current data was 0.90. In a recent systematic review of mental toughness measures, the MTI received among the highest ratings, including sufficient ratings for structural validity and internal consistency and the most positive results for hypothesis testing (Farnsworth et al., 2020).

### Statistical analysis

As the research question was to compare choking susceptible and choking non-susceptible athletes on mental toughness, the data analysis plan primarily comprised independent samples t-test on mental toughness. Prior to this analysis, assumptions (e.g., normal distribution and homogeneity of variance) would be checked. If assumptions were not upheld, a non-parametric group comparison would be employed. Furthermore, a Confirmatory Factor Analysis (CFA) of the mental toughness measure would be used to ensure that the model structure fit the current data. Finally, supplementary analysis would be employed to see if choking susceptibility may differ by factors such as gender, level of competition and experience of the sample.

Data were analyzed using SPSS 26; the factor analysis was conducted using EQS 6.4. The procedure in the current analyses that required the largest sample size was the CFA of the MTI. Tabachnick and Fidell (2021) suggest that sample sizes of over 300 are adequate when communalities in the data are high, there are a small number of factors, and at least four items for each factor, all of which were present in the current data. A power analysis using G*Power for a two tailed Mann Whitney U with an alpha of 0.05 and power of 0.95 and moderate effect size suggests a total sample size of 220. Using these criteria, our current sample size of 415 was adequate for the CFA and all subsequent analyses.

## Results

Individuals in the current sample who scored over the 75th percentile on 2 out of the 3 choking susceptible questionnaires and scored over the 50th percentile on the remaining choking susceptibility questionnaire were considered choking susceptible. As there were no observations at the 75th percentile cut-off within our data, the nearest one (73rd percentile) was used for our analyses. Out of the sample, 16% (*n* = 67) were considered choking susceptible and 84% (*n* = 348) were choking non-susceptible. Table 1 summarizes these samples by demographic characteristics.

**Table 1:** Participant descriptives.

	Choking susceptible	Choking non-susceptible
*n*	67	348
Mean Age	35.76	41.62
Male	47%	55%
Caucasian	67%	63%
Competitive	62%	47%
Starters	46%	46%

### Participant descriptives

A confirmatory factor analysis was conducted on the MTI to determine if the current data fit to the one factor model. Table 2 gives the descriptive statistics and correlations among the eight MTI items. The data upheld the assumptions of normal distribution and absence of multicollinearity (e.g., correlations >0.90). However, the normalized Mardia’s coefficient indicated multivariate kurtosis, so the robust goodness of fit indicators were interpreted. The chi-square for the model was significant [χ^2^_(20)_ = 77.10, *p* < 0.001], but this is known to be influenced by large sample size. Other goodness of fit indicators showed good fit of the data to the model, CFI = 0.95, IFI – 0.96, RMSEA = 0.08. Acceptable criteria for good fit are >0.95 for the CFI and IFI and < 0.08 for the RMSEA. All eight MTI items loaded significantly on the global MTI factor. The Cronbach’s alpha for this factor was 0.90.

**Table 2:** Correlation matrix and descriptive statistics of MTI items.

Item	MTI1	MTI2	MTI3	MTI4	MTI5	MTI6	MTI7	MTI8
MTI2	0.65^*^							
MTI3	0.56^*^	0.60^*^						
MTI4	0.53^*^	0.55^*^	0.56^*^					
MTI5	0.53^*^	0.52^*^	0.60^*^	0.66^*^				
MTI6	0.46^*^	0.45^*^	0.48^*^	0.43^*^	0.51^*^			
MTI7	0.54^*^	0.53^*^	0.47^*^	0.57^*^	0.56^*^	0.55^*^		
MTI8	0.42^*^	0.43^*^	0.46^*^	0.43^*^	0.46^*^	0.59^*^	0.55^*^	---
*M* *SD*	5.52 1.31	5.29 1.31	5.18 1.35	5.62 1.33	5.55 1.34	5.40 1.25	5.55 1.21	5.58 1.30

^*^p < 0.001.

Correlations between the MTI and the choking susceptibility scales revealed that mental toughness was uncorrelated to self-consciousness (*r* = 0.03), but significantly correlated to sport anxiety (*r* = −0.31) and the differential coping score (*r* = −0.18) in the expected directions based on this protocol. Among the two coping styles, the MTI was significantly correlated to avoidance coping (*r* = 0.15) but not significantly correlated to approach coping (*r* = −0.04).

The distribution of the MTI was not normal for both choking susceptible (*KS*_(67)_ = 0.12, *p* > 0.05) and non-susceptible athletes (*KS*_(348)_ = 0.08, *p* > 0.05), and a Levene’s test revealed that the MTI did not uphold the assumption of homogeneity of variance. Therefore, a Mann–Whitney U was used to examine if there was a difference in MTI scores between choking susceptible [*n* = 67; *M* = 42.94 (6.64)] and non-susceptible [*n* = 348; *M* = 43.83 (8.14)]. The result was non-significant [*U*_(415)_ = 10826.00 *p* > 0.05] with an effect size of 0.11. Therefore, no difference in MTI scores were found across the two groups of athletes classified as either choking non-susceptible or choking susceptible.

Given the heterogeneous nature of the sample, several supplementary analyses were conducted. In particular, we were interested in the potential role of gender, experience, and level of competition in the choking susceptible-mental toughness relationship. However, as noted above the data required non-parametric analyses and it is not possible to do factorial non-parametric analyses. Chi square analyses were used to examine if proportion of choking susceptible individuals differed by gender, level of experience, and competition. There was no significant difference in prevalence of choking susceptibility between males and females, or between individuals with less than (*n* = 182) and greater than 5 years experience (*n* = 233). There was a significant effect for level of competition on probability of choking susceptibility. Specifically, 12.5% of recreational athletes were choking susceptible whereas 19.8% of competitive athletes were. This difference in proportions was statistically significant [*χ^2^*_(1)_ = 4.09, *p* < 0.05, *φ* = −0.10].

## Discussion

The current study examined if athletes designated as choking susceptible as per Mesagno et al.’s (2008, 2009) protocol differed in their level of mental toughness from those designated as non-choking susceptible. Our hypotheses were not supported. No significant difference was found between those categorized as choking susceptible and choking non-susceptible on the MTI. This finding is inconsistent with anecdotal evidence and conceptual arguments (e.g., Hill et al., 2009, 2019) that suggest that choking susceptible athletes are more likely to be ‘less mentally tough’ than those who are not choking susceptible. However, the results of the current study are not necessarily inconsistent with empirical literature that has conceptualized mental toughness or examined mental toughness in relation to sport performance/performance under pressure (e.g., Gucciardi et al., 2015; Bédard Thom et al., 2021). The distinctness and inconsistencies presented here between mental toughness and choking susceptibility raise interesting questions about this relationship and call into question the validity of an assumed relationship between the two (Hill et al., 2009, 2019).

The current analyses suggest that choking susceptibility and mental toughness are distinct constructs. There were no significant differences on MTI scores between athletes who were choking susceptible and those who were not, suggesting that they come from the same population with respect to the attribute of mental toughness. Correlational analyses showed that MTI scores were uncorrelated with one of the three composite items of choking susceptibility (i.e., self-consciousness). Furthermore, its correlations with coping style and anxiety showed small and moderate effect sizes, respectively (Field, 2017). Whereas this is the first study to directly examine choking susceptibility and mental toughness, previous research has examined the associations between mental toughness and the composite constructs that comprise Mesagno et al.’s choking susceptibility protocol and found similar results. Some researchers have supported a negative relationship between mental toughness and anxiety (Schaefer et al., 2016; Kristjánsdóttir et al., 2019; Mojtahedi et al., 2023), but others have found no relationship (Cowden, 2017) and even a positive association between trait anxiety and mental toughness (Hardy et al., 2014). Whereas coping style has been found to be related to mental toughness (e.g., Poulus et al., 2020), very little literature has linked mental toughness to self-consciousness. Within the context of this literature, we would suggest that the present results provide support to the notion that choking susceptibility and mental toughness are distinct but related constructs.

It is possible that any relationship between choking susceptibility and mental toughness may be more nuanced than the present design was able to ascertain. Our sample was a diverse one with respect to experience, level of competition, and gender. While the overall finding was that choking susceptibility did not affect mental toughness with this sample, it is possible that there still may be a relationship between the two constructs, as the literature suggests. For example, in our sample, the prevalence of choking susceptibility was significantly higher in competitive athletes than recreational athletes. It is possible that there may be a significant difference in mental toughness between choking susceptible and non-susceptible athletes at different competitive levels. Furthermore, although we found no gender differences in choking susceptibility, given that there are often research gender differences in mental toughness (e.g., Nicholls and Polman, 2007; Madrigal et al., 2017), it is possible that the choking susceptibility-mental toughness relationship may differ by gender. In summary, we suggest that while we concluded that the constructs of choking susceptibility and mental toughness are separate but related, we acknowledge that it is possible that they may be related in specific samples (e.g., all competitive athletes, or all male athletes).

Furthermore, it may be that mental toughness is not related to choking susceptibility but isrelated to choking. It must be recognized that choking susceptibility is distinct from actual choking or performance under pressure, and that both of these later factors are more widely studied in sport psychology. Many of the associated factors noted in the introduction (e.g., anxiety, coping styles) are related to actual choking, not just choking susceptibility, and choking susceptibility has not yet been empirically linked to actually choking under pressure. Therefore, finding that mental toughness is not related to choking susceptibility may be mutually exclusive of any relationship between mental toughness and choking under pressure. This would be consistent with studies like, Hill et al. (2009, 2010), Bell et al. (2013) as well as Gucciardi et al. (2016) and Giles et al. (2018), which have suggested that mental toughness may influence performance under pressure. Again, we would suggest that there is much research to be done in this area, particularly with respect to the construct validity of choking susceptibility.

Finally, our findings of relatively minimal overlap between choking susceptibility and mental toughness may have serious implications for the construct validity of choking susceptibility, which is the newer and less well supported of the two constructs. The choking susceptibility protocol by Mesagno et al. (2008, 2009) is still in its infancy; it is unknown whether the protocol can accurately predict choking behaviors. The protocol consists of self-consciousness, trait anxiety, and coping style inventories to measure choking susceptibility. These psychological inventories have been linked to performance and ultimately choking under pressure (Mesagno, 2006). However, Mesagno admits that other factors may also influence choking susceptibility, such as introversion (Anshel, 1997). Furthermore, Mesagno recognizes that choking can be viewed as a continuum, nevertheless, Mesagno deliberately made a stringent selection criterion to purposively sample participants on opposite ends of the performance under pressure experience (Mesagno, 2006).

### Limitations and future directions

In addition to the above limitations on choking susceptibility, our hypotheses may not have been supported due to the chosen mental toughness scale. Although the MTI appears to be a measure with strong psychometric properties (Gucciardi et al., 2015), there is still much work to do with respect to understanding the conceptual clarity and mechanisms of mental toughness, for example its antecedents and outcomes and how practitioners, coaches and athletes can develop this quality over time. Furthermore, we did not ask participants’ additional information about their sport such as time spent practicing or number of competitions. We recognize that these variables and potentially others could have affected the results. Lastly, as noted above, the choking susceptibility protocol by Mesagno et al. (2008, 2009) is still in its infancy within the realm of investigating connections with performance tendencies under pressure. The protocol has not yet confirmed that it can accurately predict choking behavior in individuals. Therefore, if we were to measure performance in the current study, the results may not necessarily have given us practical data. Finally, there are potential limitations to a cross-sectional design, and online survey, as the current design employed. We acknowledge that such design features may have affected the representativeness of the sample as well as potential response biases in ways that other designs may minimize.

Future research should determine whether the choking susceptibility protocol can successfully predict choking under pressure in athletes while comparing performance under different levels of pressure. The causes and characteristics that may predispose athletes to choking can help sport psychologists prevent a possible choke. We believe that this is essential prior to examining if choking susceptible and choking resistant individuals differ on variables such as mental toughness.

## Data availability statement

The raw data supporting the conclusions of this article will be made available by the authors, without undue reservation.

## Ethics statement

The studies involving humans were approved by Brock University Research Ethics Board. The studies were conducted in accordance with the local legislation and institutional requirements. The participants provided their written informed consent to participate in this study.

## Author contributions

BT: Writing – review & editing, Writing – original draft, Investigation, Data curation. MB: Writing – review & editing, Writing – original draft, Methodology. PS: Writing – original draft, Writing – review & editing, Supervision, Formal analysis, Conceptualization.

## Footnotes

^1^http://www.danielgucciardi.com.au/questionnaires.html
